# Evaluation of c-KIT protein expression in canine mammary tumors

**DOI:** 10.1186/1753-6561-7-S2-P63

**Published:** 2013-04-04

**Authors:** Rosana CL Salvador, Talita MM Raposo, Carlos E Fonseca-Alves, Erika M Terra, Giovanna R Varallo, Renée Laufer-Amorim

**Affiliations:** 1 Department of Veterinary Clinic and Surgery, School of Agricultural Sciences and Veterinary, UNESP - Universidade Estadual Paulista, Jaboticabal, SP, Brazil; 2 Department of Veterinary Clinic, School of Veterinary Medicine and Animal Science, UNESP - Universidade Estadual Paulista, Botucatu, SP, Brazil

## Background

c-KIT is a proto-oncogene that synthesizes a tyrosine kinase protein responsible for cell growth. Higher expression of c-KIT protein in women normal breasts correlates with differentiation status of normal breast epithelium, while loss of c-KIT expression is observed during progression or malignant transformation of mammary epithelium. In bitches the role of this tyrosine kinase receptor has not been established. The aim of this study was to identify c-KIT protein expression in canine breast tissue and compared results with c-KIT expression status in human breast tissue.

## Materials and methods

c-KIT protein expression was assessed by immunohistochemistry in 26 canine mammary tumors samples and compared with normal mammary tissue, non-metastatic and metastatic mammary carcinomas. c-KIT expression was evaluated according to its pattern, as established in canine mast cell tumors (KIT I: perimembranous, KIT II: focal cytoplasmic and KIT III: diffuse cytoplasmic). Fisher exact test was used to determine the association between the categorical variables.

## Results

Metastatic carcinoma were correlated to pattern KIT II, non-metastatic carcinoma to pattern KIT I and normal breast to pattern KIT III (p<0,05).

## Conclusion

Our results differ from c-KIT protein profiles and relation to prognosis reported in canine mast cell tumors, since normal canine breast tissue had a KIT III pattern, related to worst prognosis in mast cell tumors. However, c-Kit pattern is not described in human breast cancer and correlated to prognosis.

## Financial support

FAPESP.

